# Integration of transcriptome and metabolome analyses reveals sorghum roots responding to cadmium stress through regulation of the flavonoid biosynthesis pathway

**DOI:** 10.3389/fpls.2023.1144265

**Published:** 2023-02-23

**Authors:** Zhiyin Jiao, Yannan Shi, Jinping Wang, Zhifang Wang, Xing Zhang, Xinyue Jia, Qi Du, Jingtian Niu, Bocheng Liu, Ruiheng Du, Guisu Ji, Junfeng Cao, Peng Lv

**Affiliations:** ^1^ Institute of Millet Crops, Hebei Academy of Agriculture and Forestry Sciences/ Hebei Branch of National Sorghum Improvement center/ Key Laboratory of Genetic Improvement and Utilization for Featured Coarse Cereals (Co-construction by Ministry and Province), Ministry of Agriculture and Rural Affairs/ Key Laboratory of Minor Cereal Crops of Hebei Province, Shijiazhuang, China; ^2^ Key Laboratory of Urban Agriculture (South), Ministry of Agriculture, Plant Biotechnology Research Center, Fudan-SJTU-Nottingham Plant Biotechnology R&D Center, School of Agriculture and Biology, Shanghai Jiao Tong University, Shanghai, China

**Keywords:** cadmium stress, sorghum bicolor, physiological, transcriptome, metabolome, flavonoid biosynthesis

## Abstract

Cadmium (Cd) pollution is a serious threat to plant growth and human health. Although the mechanisms controlling the Cd response have been elucidated in other species, they remain unknown in *Sorghum* (*Sorghum bicolor* (L.) Moench), an important C_4_ cereal crop. Here, one-week-old sorghum seedlings were exposed to different concentrations (0, 10, 20, 50, 100, and 150 μM) of CdCl_2_ and the effects of these different concentrations on morphological responses were evaluated. Cd stress significantly decreased the activities of the enzymes peroxidase (POD), superoxide dismutase (SOD), glutathione S-transferase (GST) and catalase (CAT), and increased malondialdehyde (MDA) levels, leading to inhibition of plant height, decreases in lateral root density and plant biomass production. Based on these results, 10 μM Cd concentration was chosen for further transcription and metabolic analyses. A total of 2683 genes and 160 metabolites were found to have significant differential abundances between the control and Cd-treated groups. Multi-omics integrative analysis revealed that the flavonoid biosynthesis pathway plays a critical role in regulating Cd stress responses in sorghum. These results provide new insights into the mechanism underlying the response of sorghum to Cd.

## Introduction

Environmental pollution with heavy metals pollution, such as cadmium (Cd), lead (Pb), copper (Cu), and zinc (Zn), has rapidly increased over recent decades. This pollution not only impairs growth and development in both plants and animals, but also threatens human health, and has become a serious environmental problem (He et al., 2013; Xue et al., 2014; Mourad et al., 2021; Meng et al., 2022). Cd is highly toxic and greatly inhibits the growth and development of plant roots, stems and other organs, causing a decrease in plant yield and quality (Feng et al., 2018; Mahmud et al., 2018; Santoro et al., 2022). Remediation of cadmium-polluted soil is therefore urgent.

Hyperaccumulators are plants capable of growing in soils with high levels of contamination and able to concentrate high levels of toxins in their tissues. Cd hyperaccumulators have been used worldwide tophyto-extract the metals from the contaminated soils in an eco-friendly and green approach (Guan et al., 2019; Bai et al., 2020).

Terrestrial plants uptake Cd from the soil mainly through their roots, and during this process, the root system initially suffers from Cd stress, triggering signal transduction (Li et al., 2012; Duan et al., 2015; Abozeid et al., 2017; Meng et al., 2022). Cd toxicity in plants can lead indirectly to the formation of reactive oxygen species (ROS), which in turn leads to cellular redox imbalance, lipid peroxidation, enzyme inactivation, disruption of nutrient absorption and assimilation, and eventually leads to the death of plant cells (Gill and Tuteja, 2010; Petrov et al., 2015; Han et al., 2016). Certain plants have therefore evolved intricate Cd detoxification strategies (He et al., 2013; Yang et al., 2021). Cd can cause changes in microstructural level, subsequently altering physiological and molecular processes. In response to Cd exposure, gene expression in certain plants exhibit can change, the antioxidative system can be activated, protein abundance can be altered and certain metabolic pathways involved in Cd uptake, transport and detoxification can be activated (He et al., 2015; Corso et al., 2018; Leng et al., 2020). Such Cd response processes have been explored largely in plant species with low biomass, such as *Arabidopsis thaliana* (L.) Heynh. *(Arabidopsis*), *Sedum aalfredii* Hance (*S.alfredii*) and *S olanum nigrum* L(*S.nigrum*) (Han et al., 2016; Luo et al., 2016; Wang et al., 2022). However, a low biomass constrains the effectiveness of these plant species in phytoremediation.

Sorghum (*Sorghum bicolor* (L.) *Moench*) is a C_4_ model plant, and is widely grown as a food, feed and energy crop (Xin et al., 2008; Xiao et al., 2021; Shi et al., 2022). Previous studies have proposed sorghum for phytoremediation due to its high production of biomass, high tolerance to abiotic stresses and the relatively high Cd accumulation observed in some genotypes (Jia et al., 2017; Feng et al., 2018; Yuan et al., 2019; Xiao et al., 2021). Sorghum is able to remedicate Cd-contaminated soil, and the polluted plant tissues can be harvested and used in the production of ethanol gas, thereby preventing Cd pollution from re-entering the food chain. This means that sorghum could be a connection between phytoremediation and bioethanol production, achieving a win-win effect (Jia et al., 2017; Feng et al., 2018; Mishra et al., 2020). Thus, it is essential to understand Cd toxicity in this species, and its response to Cd. Hydroponics and pot experiments have been conducted to study the physiological responses of different sorghum varieties to Cd stress. Cd was found to have accumulated primarily in the roots of sorghum plants and to have been partially transferred to the above ground parts. Chlorophyll content and the activities of antioxidative enzymes were affected by Cd accumulation, and led to growth reduction (Bonfranceschi et al., 2009; Soudek et al., 2014; Jawad Hassan et al., 2020). Comparative transcription analysis experiments demonstrated that higher Cd accumulation depends on multilevel coordination of efficient Cd uptake and transport (Feng et al., 2018). However, there are only a few preliminary studies into the effects of Cd stress on the growth of sorghum, and the physiological and molecular mechanisms underlying the sorghum response to Cd stress remain elusive.

In recent years, multi-omics analysis technologies have been widely utilized to study the mechanisms underlying plant responses to abiotic stress. Transcriptomics and metabolomics represent powerful tools to comprehensively analyze the biological processes and their metabolic regulation in plants growing in stressful environments (Mishra et al., 2020; Feng et al., 2021). In this study, an integrated morpho-physiological analysis combined with transcriptomics and metabolomics was performed to gain insights into the molecular mechanisms underlying sorghum responses to Cd stress. We aimed to reveal the key responsive candidate genes and pathways that respond to Cd stress in sorghum and to provide new insights to inform the genetic engineering of new plants for use in phytoremediation.

## Materials and methods

### Plant material

*Sorghum* (*Sorghum bicolor*) inbred line “407B” was used in this study. Healthy seeds were immersed in distilled water and germinated in petri dishes on filter papers. Then, seedlings with uniform growth were selected and cultivated in hydroponic boxes with 1 L 1/2 Murashige and Skoog (MS) solution (pH 5.8) (Adhikari et al., 2020). All plants were cultivated in a growth room maintained at 28 ± 2°C, with a photoperiod of 16/8 (day/night). The seedlings were grown in hydroponic medium for 7 days, and then these seedlings were subjected to different concentrations of CdCl_2_ (0, 10, 20, 50, 100 and 150 μM) (AR, AladdinBio-Chem Technology Co., Ltd, Shanghai, China) for 3 days. After treatment, sorghum plants were collected, and the plant height, root length and lateral density were measured. All samples were ground in liquid nitrogen and stored at -80°C.

### Measurement of growth index and determination of Cd concentration

The plant height, root length and the number of lateral roots were immediately measured after 3 days of growth in a Cd stress environment (Jia et al., 2017). The shoots and roots were detached and used for fresh and dry weight measurements. The shoots and roots immediately weighed to obtain the fresh weight and then dried to constant weight at 80°C to obtain the dry weight.

The Cd content in the sorghum seedlings was assayed according to a method described previously with slight modifications (Xiao et al., 2021). Briefly, roots and aboveground parts of the sorghum plants were sampled and rinsed with distilled water. Samples were then dried in an oven at 80°C until constant weight and then pulverized to a fine powder by passing through a mesh (100 mesh fractions). Afterwards, the obtained ash residues were digested using the nitric-perchloric acid (9:1, v/v) wet digestion method. The amount of Cd was measured using an atomic absorption spectrophotometer (GGX-810, Haiguang Instrument Co., Ltd, Beijing, China).

### Measurement of MDA, and H_2_O_2_ levels and activities of antioxidant enzyme

Fresh roots (0.2 g) were detached from the control and Cd treatment plants. Root concentrations of the enzymes superoxide dismutase (SOD), peroxidase (POD), catalase (CAT), glutathione S-transferase (GST), and of malondialdehyde (MDA) and hydrogen peroxide (H_2_O_2_) were analyzed using a SOD assay kit (G0101W), POD assay kit (G0107W), CAT assay kit (G0105W), GST assay kit (G0208W), MDA assay kit (G0109W) and H_2_O_2_ assay kit (G0112W), respectively, following the manufacturer’s instructions (Suzhou Grace Biotechnology Co., Ltd., Suzhou, China). The assays were performed as previously described (Jia et al., 2022). Three replicates of each treatment were used in each experiment.

### Total RNA extraction, preparation of cDNA library and Illumina RNA-seq

Based on the physiological results, samples from the 0 and 10 μM CdCl_2_ treatments were selected for transcription and metabolic analysis. The roots of the treated and control sorghum seedlings were washed with sterilized distilled water, and were immediately frozen in liquid nitrogen. Total RNA was extracted using the NEBNext^®^UltraTM RNA Library Prep Kit for Illumina^®^(NEB, USA) following manufacturer’s recommendations. The purity, concentration and integrity of RNA were assessed using a NanoPhotometer^®^ spectrophotometer (IMPLEN, CA, USA), Qubit^®^ RNA Assay Kit in Qubit^®^2.0 Flurometer (Life Technologies, CA, USA) and RNA Nano 6000 Assay Kit of the Bioanalyzer 2100 system (Agilent Technologies, CA, USA), respectively (Ren et al., 2022). One μg high quality RNA per sample was then used to construct the cDNA library, and was sequenced using an Illumina HiSeq sequencing platform by Metware Biotechnology Co., Ltd. (Wuhan, China). The software fastp v 0.19.3 was used to clean and trim the original data to obtain high-quality clean reads (reads with adapters were filtered out, as were paired reads were the N content exceeded 10% of the base number of the reads, or when the number of low-quality (Q ≤ 20) bases contained in the read exceeds 50% of the bases). Clean data were then mapped to the sorghum reference genome (Sbicolor_454_v3.0.1) using the HISAT v2.1.0 software. The expression abundance of reads was quantified using the fragments per kilobase of transcript per million base pairs (FPKM) value. The differentially expressed genes (DEGs) in the control and treatment groups were screened using DESeq2 v1.22.1 (Ross Ihaka, University of Auckland, New Zealand) with a threshold of |log_2_ Fold Change | ≥1 and False Discovery Rate (FDR)< 0.05.

### Widely-targeted metabolomics analysis

The samples described above were also used in the metabolomics analysis. The extract analysis, and the identification and quantification of metabolites were performed by MetWare (Wuhan, China) using standard procedures and based on a previously published protocol (Sun et al., 2020; Ren et al., 2022). Briefly, the freeze-dried roots were crushed using a grinder (MM 400, Retsch) at 30Hz for 1.5 min. The powder was placed in 1.2 ml of 70% aqueous methanol solution (4°C) and vortexed six times, then left over night. The extracts were filtrated through a microporous membrane (0.22 μm pore size) after being centrifuged at 12,000 g for 3 min. The extracts were analyzed using an ultra-performance liquid chromatography-tandem mass spectrometry (UPLC-MS/MS) system. Analysis of metabolite data was conducted with the Analyst 1.6.3 software. Metabolites with a fold change (FC) ≥ 2 and FC ≤ 0.5, and variable importance in projection (VIP) scores > 1 were considered to be differentially expressed metabolites (DEMs).

### Statistical analysis

All the experimental data were expressed as mean ± standard error (SE). The SPSS 26.0 Program (SPSS Inc., Chicago, IL, USA) was used for one-way ANOVA analysis at a significance level of *p< 0.05*. Origin 8 (OriginLab, Northampton, Massachusetts, USA) was used for chart processing.

## Results

### Growth characteristics of plants in responding to Cd stress

**Figure 1:**
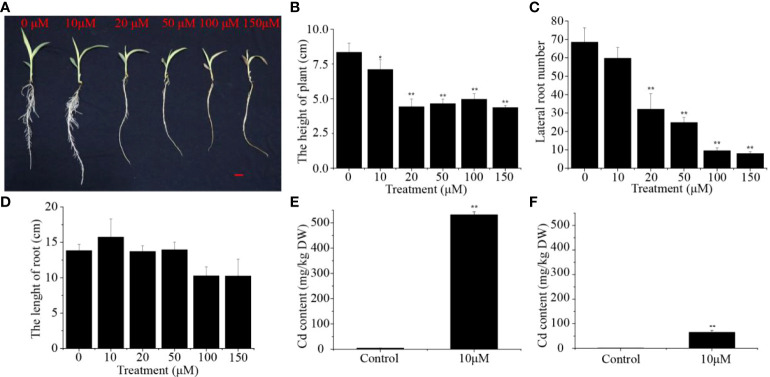
Effect of different Cd concentrations on **(A)** Phenotype, **(B)** Plant height, **(C)** The number of lateral roots and **(D)** Root length. **(E)** Cadmium concentrations in sorghum roots and **(F)** sorghum shoots under 10 μM Cd treatments. Asterisks denote significant differences (*P< 0.05; **P< 0.01). Bars = 1 cm.

To determine the potential effect of Cd stress on the growth of sorghum seedlings, the phenotypic and physiological characteristics of sorghum under Cd stress were analyzed and compared with a control group without Cd. The growth of the sorghum seedlings was markedly influenced by different concentrations of Cd, and the seedlings displayed visible poisoning symptoms including chlorosis both in leaves and roots, especially in the 150 μM Cd group (**Figure 1A**). With increasing Cd concentrations, the seedling height of the Cd-treated group was significantly reduced (14.92-47.73%) compared to that of the control group (**Figure 1B**). Seedlings treated with 10, 20, 50, 100 and 150 μM Cd had 12.85%, 53.28%, 63.87%, 86.13% and 88.32% fewer lateral roots, respectively (**Figure 1C**). Nevertheless, the length of the roots was not significantly different between these groups (**Figure 1D**). In addition, we monitored the plant fresh weight and dry weight of sorghum seedlings shoots and roots. Compared with the control, the fresh and dry weight of the aboveground and underground parts of sorghum seedlings under different cadmium concentrations were significantly reduced (**Supplementary Figure S1**). The seedlings treated with 10 μM Cd showed the minimum reduction in plant growth, and this concentration was chosen for further analysis. We calculated and compared the Cd concentrations in the shoot and root organs. We found that the Cd levels in the roots were more than 8.17 times higher than those in the shoots (**Figures 1E, F**). Taken together, these results suggest that Cd stress can negatively affect plant height and lateral root density in sorghum.

### Effect of Cd on the antioxidative enzymes in sorghum root

**Figure 2:**
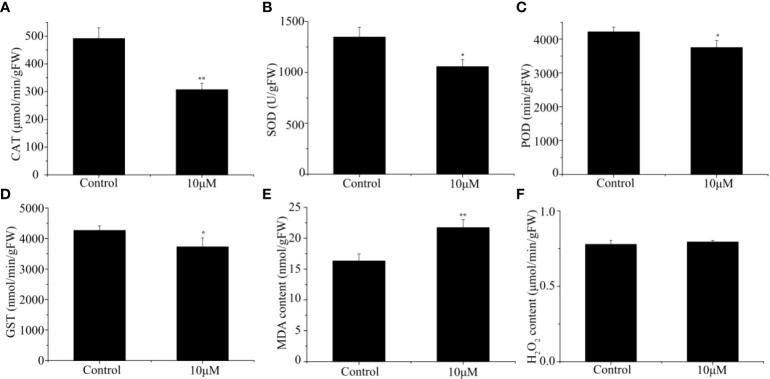
Effect of different Cd concentrations on **(A)** CAT activity, **(B)** SOD activity, **(C)** POD activity, **(D)** GST activity, **(E)** MDA content and **(F)** H_2_O_2_ content under 0 and 10 μM Cd treatments. Data are means ± SE (n = 3). Asterisks denote significant differences (*P< 0.05; **P< 0.01).

In order to further explore the effect of Cd treatment on sorghum, we analyzed the activities of antioxidative enzymes in the roots of sorghum seedlings. Under Cd stress, the activities of the tested antioxidative enzymes in the sorghum were all inhibited by varying degrees. The activity of CAT in sorghum seedlings following Cd treatment group was 37.57% lower than of the control group (**Figure 2A**). Similarly, following Cd treatment, the SOD, POD and GST activities decreased by 21.56%, 10.99% and 12.59%, respectively, compared with those in the control group (**Figures 2B–D**). We also determined the MDA and H_2_O_2_ content in the sorghum roots. The level of MDA in Cd-treated plants was 1.33-fold that in control plants (**Figure 2E**), However, the H_2_O_2_ content in the roots was not significantly different, from that in the control, although it exhibited a slight increase with increasing Cd levels (**Figure 2F**). In general, Cd exposure causes a severe oxidative injury in plant tissues.

### Transcriptome analysis of sorghum roots under Cd stress

We then wanted to investigate the potential molecular mechanism underlying the phenotypes observed in sorghum seedlings under Cd stress. Cd is a kind of trace heavy metal, which can produce toxic effects on plants and human body at low doses (Haider et al., 2021). Previous studies have focused on the harm of high concentrations to plants (Leng et al., 2020; Yang et al., 2021; Wang et al., 2022), while the low dose of Cd in soil is also a common problem to be solved in human life and crop production (Kubier et al., 2019; Wang et al., 2019). Combined with our physiological data and the results of the study on cadmium tolerance in sorghum (Jia et al., 2017; Feng et al., 2018), 10μM is a suitable concentration choice, so we chose this concentration for further research Samples from the 0 and 10 μM Cd treatment groups were selected as study materials for the transcriptome and metabolome analyses. In total, 6 libraries (control and Cd treated plants, each with three biological replicates) were generated and 407,987,342 raw data were sequenced. After removing low-quality reads, a total of 397,496,580 clean reads remained. The Q20, Q30 and GC content values of the clean reads were 97.93 – 98.32%, 94.14 – 95.02% and 54.18 – 55.42%, respectively (**Supplementary Table S1**), indicating that the transcriptome sequencing results were reliable and could be used for further DEG analysis.

**Figure 3:**
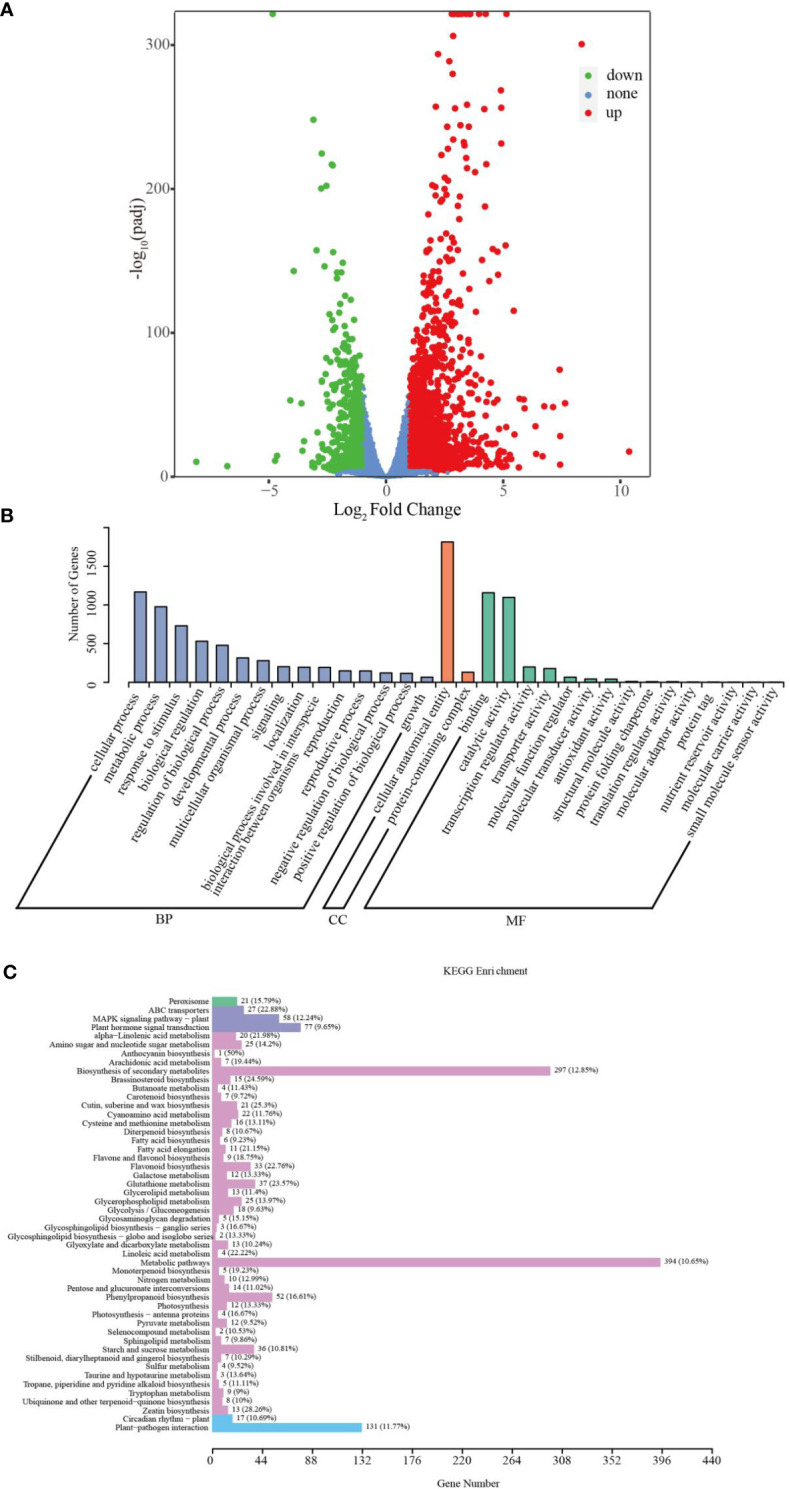
Transcriptomic analysis of sorghum roots under 0 and 10 μM Cd treatments. **(A)** Volcano plots of DEGs. **(B)** GO classification of DEGs. **(C)** KEGG enrichment of DEGs.

The percentage of reads that could be mapped to the sorghum reference genome was 91.86 – 93.62%, among which 89.02 – 90.76% were uniquely mapped (**Supplementary Table S1**), indicating that the selected reference genome was suitable. The correlation analysis revealed that treatment groups had appropriate intra - group biological repeatability (**Supplementary Figure S2A**). Principal component analysis (PCA) analysis demonstrated that the samples within the control and treatment groups clustered together in their respective groups, and the expression of gene clusters between the control and Cd treatment groups was clearly distinguished (**Supplementary Figure S2B**). Based on standard cut-offs (|fold change| > 2 and corrected p-value< 0.05), we identified the differentially expressed genes (DEGs) in sorghum roots in response to Cd stress. A total of 2683 DEGs (1812 up- and 871 down-regulated) were characterized in the control and Cd treatments (**Figure 3A**; **Supplementary Table S2**).

### GO and KEGG pathway analysis of DEGs

To assess the biological functions of DEGs in sorghum roots under Cd treatment, Gene Ontology (GO) and KEGG enrichment analyses of DEGs were carried out. GO classification analysis showed that the DEGs could be predominantly classified into three ontologies concerning biological process (BP), molecular function (MF) and cellular component (CC), as shown in **Figure 3B**. Theses DEGs could be further divided into 32 functional GO terms. In the BP group, the most abundant terms were “cellular process”, “metabolic process” and “response to stimulus”. In the MF category, “binding”, “catalytic activity”, “transcription regulator activity” and “transporter activity” were the most highly represented terms; and in the CC processes, the most abundant term was “cellular anatomical entity” (**Figure 3B**; **Supplementary Table S3**). Additionally, KEGG analysis assigned these DEGs to 120 KEGG pathways (**Supplementary Table S4**), of which the top 50 are given in **Figure 3C**. In particular, “Biosynthesis of secondary metabolites” (ko01110), “Glutathione (GSH) metabolism” (ko00480), “Flavonoid biosynthesis” (ko00941), “ATP-binding cassette (ABC) transporters” (ko02010), “Phenylpropanoid biosynthesis” (ko00940), “Metabolic pathways” (ko01100) and “Flavone and flavonol biosynthesis” (ko00944) were significantly enriched. These results indicate that sorghum roots can effectively enhance GO terms such as metabolic process, response to stimulus, catalytic activity or transporter activity to improve the Cd tolerance of plants.

### Metabolomic analysis of sorghum root under Cd stress

**Figure 4:**
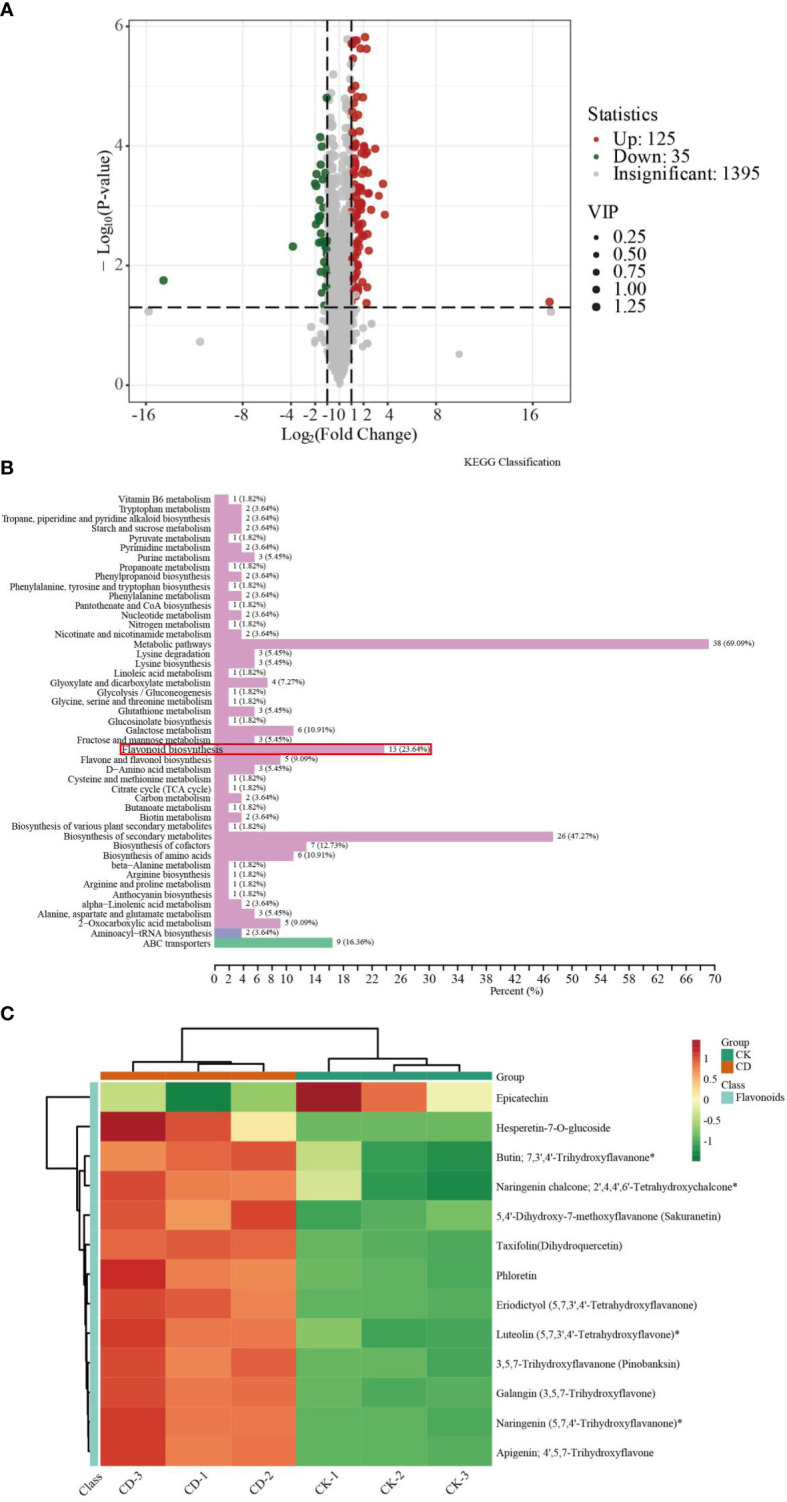
Metabolite analysis of sorghum roots under 0 and 10 μM Cd treatments. **(A)** Volcano plots of DEMs. **(B)** KEGG classification of DEMs. **(C)** Heat map of DEGs in flavonoid biosynthesis pathway.

To further elucidate the responses of sorghum roots to Cd stress, we conducted widely targeted metabolomics analyses using an UPLC-MS/MS system. PCA and orthogonal projections to latent structures-discriminant analysis (OPLS-DA) score plot results showed that different samples under the same treatment conditions clustered together, whereas samples from different treatment groups were significantly separated (**Supplementary Figures S3A, B**). The correlation analysis of metabolites in different samples showed biological repeatability between groups (**Supplementary Figure S3C**). In addition, these DEMs fell into 12 categories (**Supplementary Figure S3D**). The most abundant classes were flavonoids (20.84%), followed by phenolic acids (17.04%), amino acids and their derivatives (12.6%), alkaloids (10.87%), lipids (10.48%), others (9.13%), organic acids (6.56%), nucleotides and their derivatives (4.69%), lignans and coumarins (3.47%), terpenoids (3.02%), quinones (1.09%) and tannins (0.19%). A total of 1555 metabolites was detected (**Supplementary Table S5**). We screened the DEMs based on the selection criteria listed in the methods section above. The abundance of 160 metabolites changed differentially between the two treatment groups, and included 35 down-regulated and 125 up-regulated metabolites (**Figure 4A**). Detailed information regarding the 160 metabolites is given in **Supplementary Table S6**.

### KEGG enrichment analysis of DEMs

To further confirm the key metabolic pathways related to sorghum root responses to Cd toxicity, we conducted KEGG enrichment pathways analysis. As shown in **Figure 4B** and **Supplementary Table S7**, KEGG pathway enrichment analysis indicated that these DEMs could be catagorized into 47 species, the most significant of which were metabolic pathways (38), biosynthesis of secondary metabolites (26), followed by flavonoid biosynthesis (13), ABC transporters (9) and others. The results of this analysis suggest that these metabolites could play a vital role in the response to Cd stress in sorghum roots.

We then constructed a heat map of flavonoid biosynthesis metabolite expression to better visualize the expression patterns of related metabolites in the control vs Cd-treated comparison groups. Thirteen DEMs including hesperetin-7-O-glucoside, butin; 7,3’,4’-trihydroxyflavanone, naringenin chalcone; 2’,4,4’,6’-tetrahydroxychalcone, 5,4’-dihydroxy-7-methoxyflavanone (sakuranetin), taxifolin (dihydroquercetin), phloretin, eriodictyol (5,7,3’,4’-tetrahydroxyflavanone), luteolin (5,7,3’,4’-tetrahydroxyflavone), 3,5,7-trihydroxyflavanone (pinobanksin), galangin (3,5,7-trihydroxyflavone), naringenin (5,7,4’-trihydroxyflavanone) and apigenin; 4’,5,7-trihydroxyflavone, were found to be related to flavonoid biosynthesis (**Figure 4C**). These results were essentially consistent with the transcription results.

### Integrated analysis of the transcriptome and metabolome analyses

**Figure 5:**
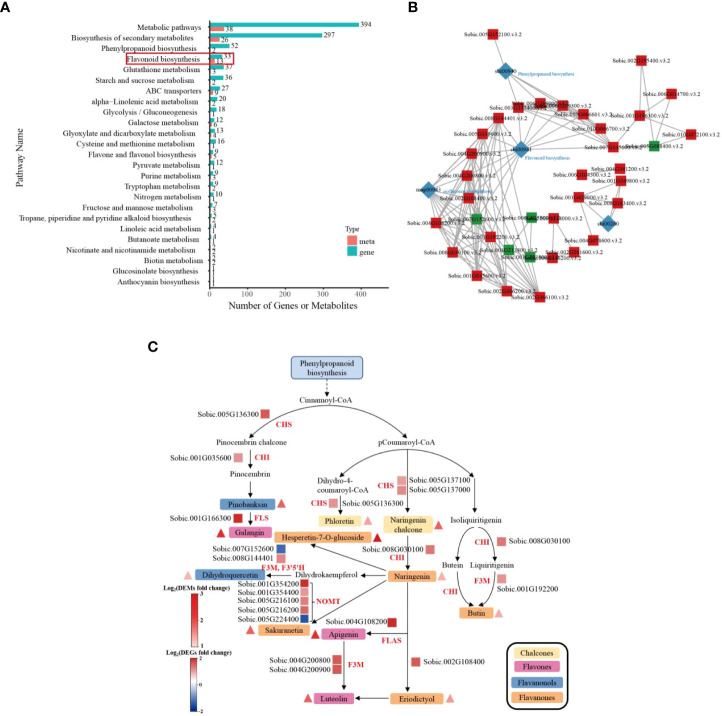
Transcriptomic and metabolomic analysis of sorghum roots under 0 and 10 μM Cd treatments. **(A)** KEGG enrichment analysis histogram of the DEGs and DEMs of sorghum roots. **(B)** KGML network of DEGs and DEMs. The square represents the gene or gene product (red: up-regulated, green: down-regulated), and the diamond represents the pathway. **(C)** Parallel displays of DEGs and DEMs involved in flavonoid biosynthesis in sorghum roots in response to Cd stress. The triangle and rectangle represent the metabolites and genes regulated under Cd treatment, respectively (red, up-regulation; blue, down-regulation). The types of metabolites are distinguished by long rectangular boxes with different colors (CHS, chalcone synthase; CHI, chalcone-flavonone isomerase; FLS, flavonol synthase; F3M, flavonoid 3’-monooxygenase; F3’5’H, flavonoid 3′,5′-hydroxylase; NOMT, naringenin 7-O-methyltransferase; FLAS, flavonol synthase II).

To understand the mechanisms underlying the response of sorghum to Cd stress, we then conducted an analysis of the relationship between DEMs and DEGs. The results of KEGG pathway enrichment analysis gave the number of differential metabolites and differential genes enriched in the flavonoid biosynthesis pathway, ABC transporters, flavone and flavonol biosynthesis pathway and other pathways (**Figure 5A**; **Supplementary Table S8**). As shown in **Figure 5B** and **Supplementary Table S9**, the co-expression network analysis of DEGs and DEMs in control and Cd-treated groups showed that the terms were mainly enriched in flavonoid biosynthesis-sorghum bicolor (sbi00941), isoflavonoid biosynthesis (map00943), and phenylpropanoid biosynthesis-sorghum bicolor (sbi00940 and sbi00260). The results suggest that the flavonoid biosynthesis pathway might have a crucial role in the responses of sorghum roots to Cd stress.

To further investigate the effect of Cd stress on of genes and metabolites related to flavonoid biosynthesis in sorghum roots, the DEGs and DEMs related to flavonoid biosynthesis pathway were analyzed. The key genes in the flavonoid biosynthesis pathway, including NOMT (naringenin 7-O-methyltransferase), F3’5’H (flavonoid 3′,5′-hydroxylase), CHS (chalcone synthase), CHI (chalcone-flavonone isomerase) and F3M (flavonoid 3’-monooxygenase), were found to be upregulated in the Cd-treated group. The increase in naringenin coincided with the upregulation of genes encoding CHS (chalcone synthase) and CHI (chalcone-flavonone isomerase). Moreover, metabolites associated with flavonoid biosynthesis, including hesperetin-7-O-glucoside, accumulated significantly in sorghum under Cd treatment (**Figure 5C**; **Supplementary Table S10**). The integrated analysis results demonstrated that the DEGs and DEMs related to the flavonoid biosynthesis pathway were a response to Cd stress in sorghum roots. Sorghum therefore has a complex network of gene and metabolite regulation to improve its resistance to Cd.

## Discussion

### Effect of Cd stress on the growth phenotype and physiology of sorghum

Cd, a toxic and nonessential heavy metal, can alter plant physiological and biochemical parameters, inhibit plant growth and hinder plant yield (Li et al., 2019; Wang et al., 2022). This study explored the effects of different concentrations of Cd on the growth of sorghum seedlings. The experimental results demonstrated the negative impacts of Cd on sorghum root growth and on the enzyme activities in the roots. Sorghum leaves curled and the tips became brownish under Cd stress, and consistent with previous studies, Cd induced chlorosis and a dwarf phenotype in these plants (Roy et al., 2016). In addition, a dose-dependent decline in shoot height, number of lateral root and plant biomass production, with high levels of Cd accumulating in both roots and shoots (**Figure 1**). Previous studies have demonstrated that exposure of Pb in *T. pratense* and maize seedlings under VI conditions has toxic effects in a concentration-dependent manner, and that Pb is more toxic at high concentrations (Adhikari et al., 2020; Meng et al., 2022). We found that the growth inhibition of sorghum seedlings treated with high Cd concentrations was greater than that under lower Cd concentrations (**Figures 1B, C**). A similar phenomenon has been repeatedly observed in many plant species under Cd stress (Chen et al., 2020; Khan et al., 2020). Soudek et al. (2014) found that 100-500 μM Cd treated sorghum plants after 7 days, 14 days, 28 days already have a reduction in the growth of the root system. However, our findings differ from this to some degree. Different Cd concentrations higher than 10 μM significantly reduced the number of lateral roots in sorghum seedlings in our experiments, but had no effect on root length (**Figure 1D**). It has been suggested that Cd affects plant root development in a time and concentration-dependent manner (Abozeid et al., 2017; Zhan et al., 2017; Cao et al., 2019), so we speculate that the Cd treatment in our experiment does not affect sorghum root length due to the short treatment time. We also observed a poisoned phenotype in the leaves and roots of young sorghum seedlings under Cd stress, especially at elevated concentrations (**Figure 1A**). Published studies have demonstrated that Cd has a toxic effect on plant leaves because it damages the ultrastructure of chloroplasts, so that the plant leaves appear yellowish (Liu et al., 2019; Chen et al., 2021). Furthermore, the accumulation of Cd in plants also damages the mitochondria and other organelles, significantly reducing the efficiency of photosynthesis and therefore the energy supply, further inhibiting plant growth (Minkina et al., 2018; Chen et al., 2020; Yadav et al., 2021; Wang et al., 2022). In other words, the toxic effect of Cd on plants is concentration-dependent, and Cd has a greater impact on plant growth at elevated concentrations.

Heavy metal stress leads to the production of reactive oxygen species (ROS) in plants (Adhikari et al., 2020). Plants are able to protect themselves from oxidative damage caused by stress through enzymatic systems, with the POD and CAT enzymes, for example, responsible for the conversion of H_2_O_2_ to water. Another ROS scavenging enzyme, SOD, also plays an essential role in mitigating oxidative stress. SOD can modulate the amount of superoxide radicals $\left({\text{O}}_{2}^{-}\right)$ , which involves the dismutation of $\left({\text{O}}_{2}^{-}\right)$ into H_2_O_2_ and O_2_ under stress conditions (Liu et al., 2008; Zornoza et al., 2010). The main function of GST is considered to be detoxification, and this enzyme also plays a major role in ROS scavenging (Dixon et al., 2002; Zagorchev et al., 2013). In the current work, the activities of SOD, POD, CAT and GST in the roots of sorghum all decreased following treatment of the plants with 10 μM Cd (**Figure 2**). This suggests that the activities of these antioxidant enzymes in sorghum roots are suppressed under 10 μM Cd stress. Previous studies have verified that Cd-induced oxidative stress of membrane lipids, harms the thylakoid membrane structure (He et al., 2017; Saleem et al., 2020). Our results show that levels of MDA in the group treated with Cd were significantly higher than those in the control group (**Figure 2E**). We also found that there was no significant difference in H_2_O_2_ content between the control and Cd-treated groups, although there was a slight increase. Previous studies have shown that the accumulation of H_2_O_2_ is time-and concentration-dependent (Lv et al., 2017), so we speculate that this may be the reason why H_2_O_2_ has not changed significantly in the current experiment.(**Figure 2F**). From our physiological data, 10µM Cd caused damage to the growth of the sorghum seedlings. Cd stress thus caused the sorghum seedlings to suffer from oxidative stress, which then impacted the growth of the seedlings.

### The transcriptomic and metabolomic analyses of sorghum roots in response to Cd toxicity

To further investigate the physiological and molecular mechanisms of the response in sorghum to Cd, the metabolomic and transcriptomic analyses of sorghum roots exposed to Cd were adopted. From the transcriptomic data, we identified 2683 DEGs among the treatment groups (**Figure 3A**, **Supplementary Table S2**). These DEGs were involved in certain biological pathways, including metabolic process, response to stimulus, catalytic activity, and transporter activity (**Figure 3B**), indicating that the sorghum roots could efficiently activate the defense system and induce the expression of stress-related genes to when subjected to Cd toxicity. Previous studies have shown that Cd stress enhances the levels of various metabolites related to amino acid, carbohydrate, and nucleotide biosynthesis pathways *via* regulation of the expression of DEGs (Singh et al., 2016; Wang et al., 2022). We identified 160 DEMs (**Figure 4A**). In addition, we found that some metabolic pathways were induced markedly in sorghum roots under Cd stress, including flavonoid biosynthesis, ABC transporters, flavone and flavonol biosynthesis, and other pathways (**Figure 4B**; **Supplementary Table S7**). Transcriptome and cDNA-microarray studies have found that ABC transporters were involved in the tolerance and detoxification of heavy metals in plants, and have identified a large number of ABC transporter genes that are induced or inhibited under Cd treatments (Bovet et al., 2005; Li et al., 2022a). Moreover, ABC transporters in transgenic plants have higher Cd tolerance compared to the wild type, and are thought to increase their tolerance of Cd stress by modifying the transport and distribution of Cd, for example by exporting Cd from the root cells or by forming Cd conjugates and subsequently sequestrated Cd in vacuoles *via* ABC transporters for detoxification (Wojas et al., 2009; Park et al., 2012; Brunetti et al., 2015; Wang et al., 2017; Fu et al., 2019). Winter et al. (2015) reported that the ABC transporter can assist the transport of Cd to the xylem through the ectoplasmic pathway to transport and fix Cd. Our results confirm the function of ABC transporters in cadmium stress, and also verify the reliability of our omic data. Previous studies have also shown that phenylpropanoid biosynthesis may play an important role in Cd resistance (Feng et al., 2018; Quan et al., 2020; Zhu et al., 2020). Furthermore, KEGG pathway analysis found that the phenylpropanoid biosynthesis pathway showed significant differences between two sorghum genotypes (one high-Cd accumulation and one low-Cd accumulation) upon Cd stress (Feng et al., 2018). In our study, the phenylpropanoid biosynthesis pathway was enriched in the KEGG pathways of the DEGs, and in the co-expression network analysis of DEGs and DEMs. However, the genotype of Cd accumulation in 407B could not be determined by our study, and needs further research.

By combining the transcriptomic and metabolic results, we identified that the flavonoid biosynthesis pathway was a crucial pathway in Cd stress responses in sorghum seedlings, and that there were several DEGs and DEMs in the sorghum roots activated in the response to Cd stress. We have provided a more complete molecular outline of sorghum resistance to Cd stress (**Figure 5C**, **Supplementary Table S10**). Flavonoids are a ubiquitous group of polyphenol compounds, belonging to non-enzymatic antioxidants and composed of flavones, flavonols, flavanones, isoflavones and anthocyanins (Nijveldt et al., 2001; Sun et al., 2020). It has been demonstrated that flavonoids play an important role in tolerance to environmental stresses, such as heavy metals and drought in plants (Meng et al., 2022). The accumulation of flavonoids is a hallmark of plant stress, and the flavonoids synthesis can be activated under oxidative injury. Recent studies have shown that flavonoids not only have apparent roles in stress protection, but also participate in regulating auxin transport and altering plant growth (Li et al., 2022b; Winkel et al., 2022). In our study, certain flavonoid metabolites accumulated under Cd stress (**Figure 5**). CHS catalyzes the condensation of one molecule of 4- coumaroyl-CoA and three molecules of malonyl-CoA to naringenin chalcone, which is the substrate for CHI and which is converted to naringenin, CHS and CHI are the key rate-limiting enzymes in the flavonoid biosynthesis pathway (Rani et al., 2012; Liu et al., 2021). Wang et al. (2021) have shown that there were devoid of tricin-lignin in the cell walls and depleted of flavones in *CHS*- and *CHI-*deficient rice mutants. The biosynthesis of lignin affected the growth of plant seedlings and altered plant morpho-physiological under Cd treatment (Feng et al., 2018). We screened the CHS and CHI genes, which were up-regulated following Cd treatment. We observed an accumulation of the substances involved in the flavonoid biosynthesis pathway, including naringenin chalcone and naringenin (**Figure 5**; **Supplementary Table S10**). Some secondary metabolites, including flavones, are mainly used in plants for chelating metal ions to confer Cd resistance in plants (Zhu et al., 2020; Li et al., 2022; Sharma et al., 2022). In this study, flavones, including galangin, apigenin and luteolin, accumulated during Cd stress, and the genes encoding these key enzymes in this pathway, such as FLS, F3M and FLAS, were upregulated following Cd treatment (**Figure 5**). Increased levels of flavonoids in plants can reduce Cd poisoning through chelation and passivation (Li et al., 2015; Cai et al., 2017). This is consistent with our experimental data. Consequently, we infer that the up-regulation of genes involved in the flavonoid synthesis pathway together with the increase in flavonoid metabolites are closely related to sorghum resistance to Cd stress. These findings confirm the importance of the flavonoid metabolic pathway, which is involved in the response of sorghum roots to Cd stress.

## Conclusion

In summary, this study investigated the effects of different concentrations of Cd on the growth and physiology of sorghum seedlings. Cd toxicity significantly inhibited plant height and lateral root development, especially at high Cd concentrations. Cd stress also altered the activities of antioxidant enzymes (SOD, POD, GST, CAT) and the concentrations of MDA. Moreover, transcriptome and metabolomics analyses revealed the mechanism underlying the response of sorghum to low concentrations (10 μM) of Cd. Transcriptomic and metabolomic analyses revealed that Cd treatment induced numerous pivotal DEGs and altered certain key metabolites enriched in the flavonoid biosynthesis pathway. Our findings provide new insights into the physiological and molecular mechanisms of sorghum responses to Cd stress. In this current study, we used only a single sorghum variety and a single Cd concentration. Future research should also investigate proteomics to further explore the subtle molecular mechanisms underlying sorghum responses to Cd at different concentration.

## Data availability statement

The datasets presented in this study can be found in online repositories. The names of the repository/repositories and accession number(s) can be found below: https://ngdc.cncb.ac.cn/gsa. accession number CRA009573.

## Author contributions

ZJ, YS, PL, and JC conceived, supervised the experiment, revised the manuscript, and coordinated the study. ZJ, JW, ZW, XZ, XJ, and QD performed research. BL, JN, RD, and GJ assisted in writing the original manuscript. All authors contributed to the article and approved the submitted version.

## Supplementary material

Supplementary Figure 1 Effect of different Cd concentrations on (A) Aboveground fresh weight, (B) Aboveground dry weight, (C) Underground fresh weight, (D) Underground dry weight.

Supplementary Figure 2 Transcriptomic differences between control and Cd-treated groups. **(A)** PCC analysis and **(B)** PCA score plot.

Supplementary Figure 3 Metabolomic differences between control and Cd-treated groups. **(A)** PCA score plot, **(B)** OPLS-DA score plot, **(C)** PCC analysis, **(D)** Classification of secondary metabolites.

Supplementary Table 1 Statistics regarding transcriptome sequencing results.

Supplementary Table 2 Summary information of DEGs.

Supplementary Table 3 GO analysis of DEGs.

Supplementary Table 4 KEGG enrichment of DEGs.

Supplementary Table 5 Information about all metabolites.

Supplementary Table 6 Information about DEMs.

Supplementary Table 7 KEGG classification of DEMs.

Supplementary Table 8 KEGG enrichment of DEGs and DEMs.

Supplementary Table 9 Co-expression network of DEGs and DEMs.

Supplementary Table 10 List of DEGs and DEMs involved in the flavonoid biosynthesis pathway.
