# Supplementary material for: Integration of transcriptome and metabolome analyses reveals sorghum roots responding to cadmium stress through regulation of the flavonoid biosynthesis pathway
Source: Front Plant Sci. 2023 Feb 23;14:1144265. doi: 10.3389/fpls.2023.1144265 (PMC9996021; doi:10.3389/fpls.2023.1144265)
Supplement: Supplementary Figure 1 — Effect of different Cd concentrations on (A) Aboveground fresh weight, (B) Aboveground dry weight, (C) Underground fresh weight, (D) Underground dry weight. [file Image_1.pdf]

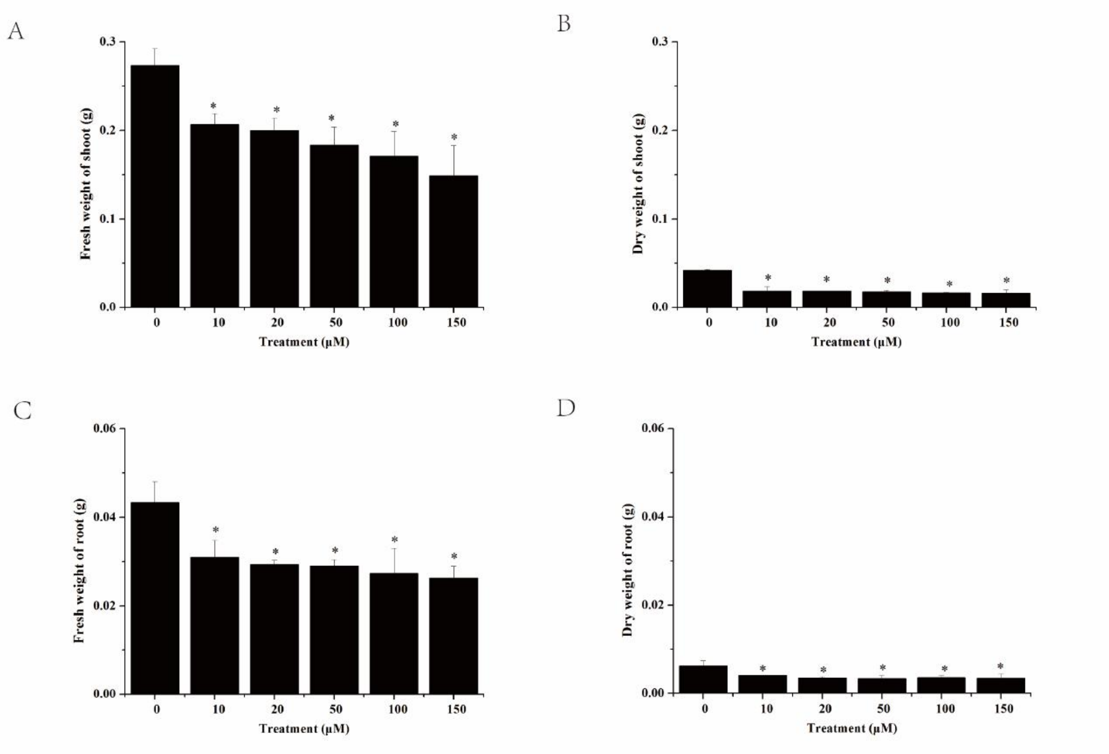

SUPPLEMENTARY FIGURE S1| Effect of different Cd concentrations on (A) Aboveground fresh weight, (B) Aboveground dry weight, (C) Underground fresh weight, (D) Underground dry weight.

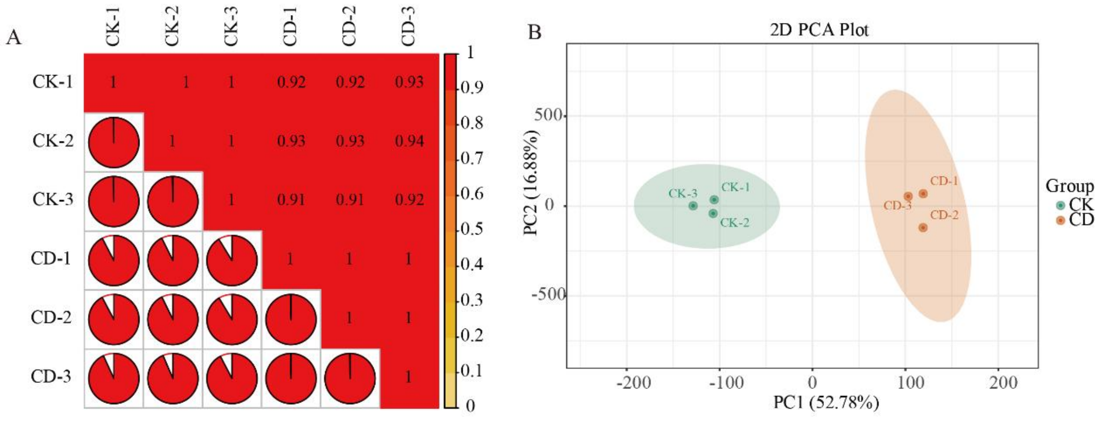

SUPPLEMENTARY FIGURE S2| Transcriptomic differences between control and Cd-treated groups. (A) PCC analysis and (B) PCA score plot.

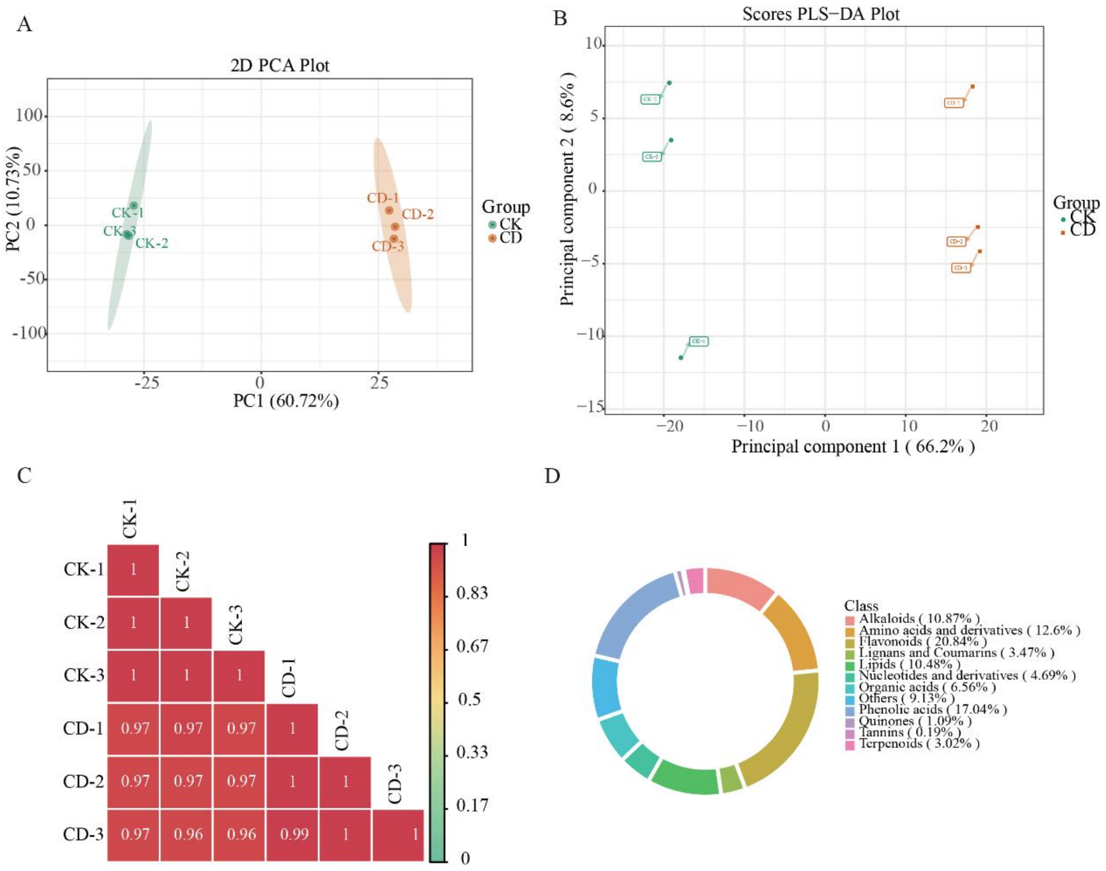

SUPPLEMENTARY FIGURE S3| Metabolomic differences between control and Cd-treated groups. (A) PCA score plot, (B) OPLS-DA score plot, (C) PCC analysis, (D) Classification of secondary metabolites.
